# Post-transplantation encapsulating peritoneal sclerosis without inflammation or radiological abnormalities

**DOI:** 10.1186/1471-2369-14-203

**Published:** 2013-09-26

**Authors:** Sayed Meelad Habib, Frank Johan Marinus Frederik Dor, Mario Richard Korte, Sander Martijn Hagen, Michiel Gerardus Henricus Betjes

**Affiliations:** 1 Department of Internal Medicine, Division of Nephrology and Transplantation, Erasmus Medical Center, D-414, P.O. Box 2040, Rotterdam 3000, CA, The Netherlands; 2 Department of Surgery, Division of Transplant Surgery, Erasmus Medical Center, Rotterdam, The Netherlands; 3 Department of Internal Medicine, Division of Nephrology, Albert Schweitzer Hospital, Dordrecht, The Netherlands

**Keywords:** Adhesiolysis, Encapsulating peritoneal sclerosis, Feces sign, Kidney transplantation, Laparotomy, Peritonectomy

## Abstract

**Background:**

Post-transplantation encapsulating peritoneal sclerosis (EPS) causing bowel obstruction has been identified as a serious complication after kidney transplantation in patients previously treated with peritoneal dialysis. Systemic inflammation and abnormalities on an abdominal computed tomography (CT) scan are important hallmarks of EPS. To our knowledge, this is the first report of a case being diagnosed with late-onset post-transplantation EPS without systemic inflammation or abnormalities on a CT scan which could only be diagnosed by laparotomy.

**Case presentation:**

A 59-year old female presented because of symptoms of bowel obstruction 33 months after kidney transplantation. The patient had a 26-month history of peritoneal dialysis before her first kidney transplantation and was treated with peritoneal dialysis for 4 years before undergoing a second kidney transplantation. Physical examination was unremarkable and laboratory tests showed no signs of systemic inflammation (C-reactive protein <1 mg/L). An abdominal CT scan did not reveal any abnormalities fitting the diagnosis of EPS, except a “feces sign”. Given the severity of the progressive symptoms, a diagnostic laparotomy was performed, visualizing a classical EPS. Total peritonectomy and enterolysis were performed, leading to restoration of peristalsis.

**Conclusion:**

EPS may occur several years after kidney transplantation in the absence of inflammation and typical radiological abnormalities. Obtaining a diagnosis of post-transplantation EPS is challenging, however, a low threshold for surgical exploration in case of high clinical suspicion and negative findings on the CT scan is mandatory.

## Background

Encapsulating peritoneal (EPS) sclerosis is a rare but potentially lethal complication of peritoneal dialysis (PD) treatment, characterized by excessive sclerotic and fibrotic thickening of the peritoneal membrane, which may eventually lead to bowel obstruction [1,2]. In recent years, EPS occurring after kidney transplantation (KTX) in patients who were previously being treated with PD (coined post-transplantation EPS) is increasingly observed [3,4]. The prevalence of post-transplantation EPS is reported to be 1-3% in PD patients undergoing KTX and is associated with significant mortality [4-6]. The prognosis of EPS can be substantially improved by treating with tamoxifen, anti-inflammatory drugs, and timely surgical intervention [7,8].

The time from KTX until development of post-transplantation EPS is typically short with the vast majority occurring within the first year after transplantation [4,9,10]. In most cases, EPS is accompanied by systemic inflammation [11]. The presence of symptoms of obstructed bowel movements and characteristic findings on computed tomography (CT) scan of the abdomen are mandatory for the clinical diagnosis of EPS [1,12]. We here report a unique case of post-transplantation EPS presenting 33 months after KTX in a patient with progressive symptoms of intestinal obstruction without systemic inflammation and absence of typical radiological abnormalities. This unusual presentation emphasizes the value of considering a diagnosis of EPS even several years after KTX. Whenever there is a high degree of suspicion for this condition in former PD patients undergoing KTX, a laparotomy by a surgeon with EPS experience is recommended.

## Case presentation

A 59-year old female was referred to our EPS center outpatient clinic because of progressive symptoms of bowel obstruction almost 33 months after KTX. The nausea, vomiting and abdominal pain predominantly occurred shortly after eating solid meals. The patient’s medical history included end stage renal disease secondary to polycystic kidney disease. She had a 26-month history of PD before her first KTX and was treated with PD for almost 4 years before undergoing her second transplantation with a kidney from a living donor. During PD treatment she had no signs of ultrafiltration failure and had experienced one uncomplicated peritonitis episode with Staphylococcus aureus.

Her symptoms had started insidiously one year after transplantation while she received a tacrolimus-based immunosuppressive regimen. Diagnostic work-up did not show any signs of inflammation with normal radiological imaging of the abdomen. Almost 23 months after her last transplantation a diagnostic laparoscopy was performed elsewhere, but no macroscopic abnormalities were reported. However, peritoneal biopsies were taken and showed signs of fibrosis without active or chronic infiltration. As the symptoms and weight loss persisted, she was referred to our clinic for a second opinion.

**Figure 1:**
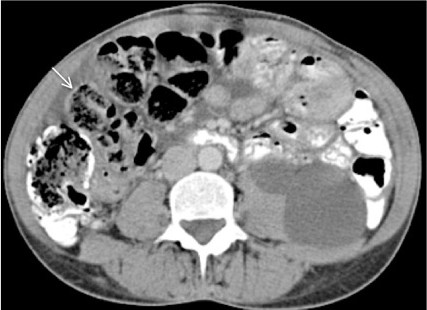
Abdominal computed tomography scan demonstrating the presence of feculent material in the small bowel (arrow) and a small amount of localized ascites.

**Figure 2:**
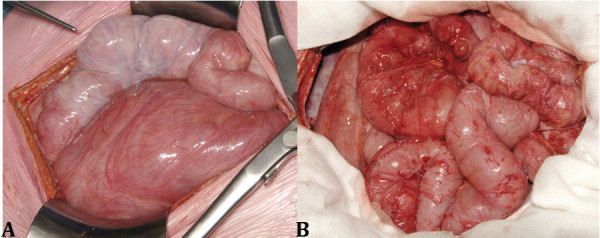
**Macroscopic appearance of EPS before and after surgery.** During the laparotomy, a classical picture of EPS was observed characterized by a thin cocoon-like sclerotic membrane encasing the small bowel **(A)**. A complete resection of the encapsulating sclerotic membrane and total enterolysis were performed, combined with removal of the thickened visceral peritoneal membrane **(B)**.

**Figure 3:**
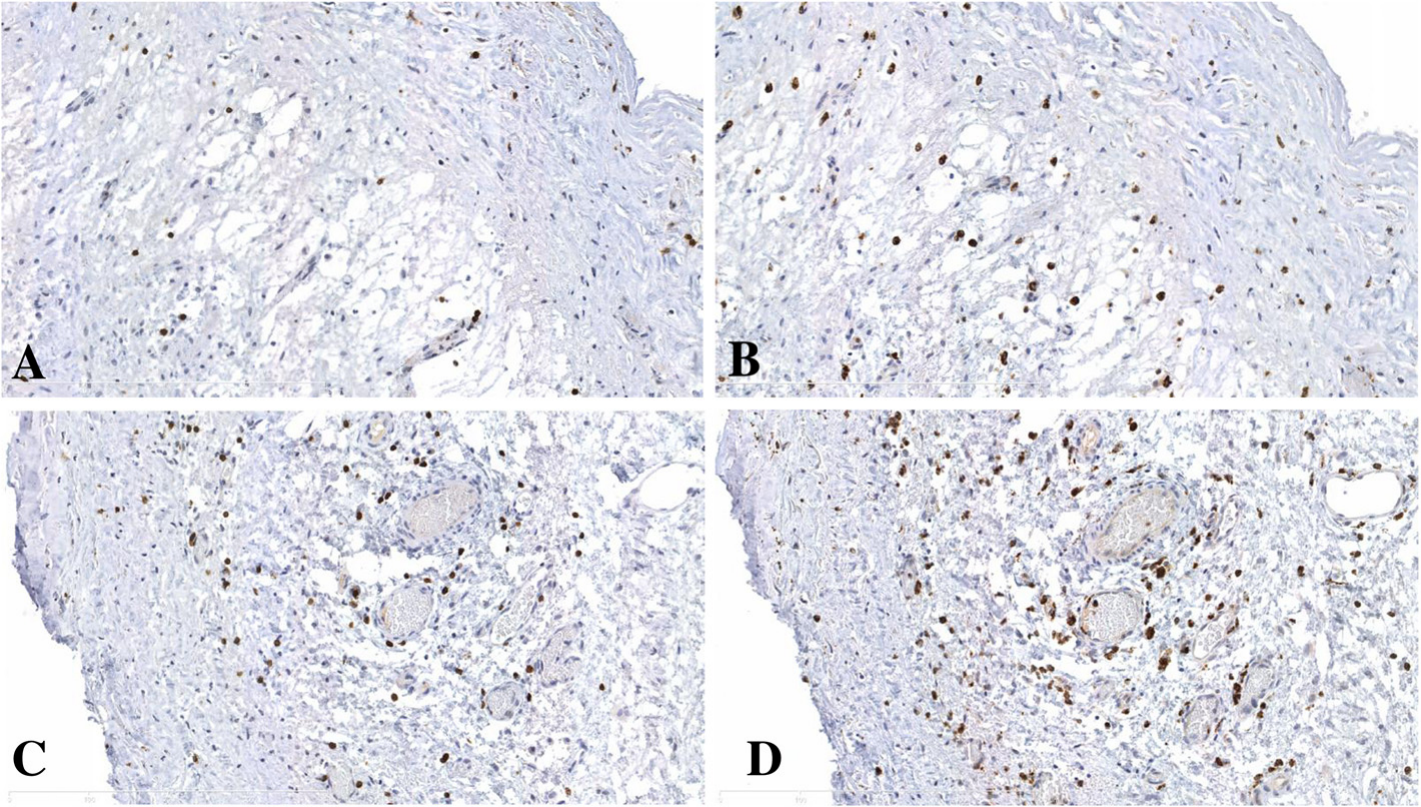
**Microscopic evaluation of the visceral peritoneal membrane revealed dense sclerosis with patchy mononuclear cell infiltration.** Immunostaining with anti-CD3 antibody **(A**,**C)** and anti-CD68 antibody **(B**,**D)**. Original magnification × 200. Scale bar represents 400 μm.

At presentation she had lost 5 kilograms of weight over the last year (BMI 19 kg/m^2^) and was unable to tolerate solid foods. No abnormalities were found at physical examination, and laboratory tests showed no signs of systemic inflammation (C-reactive protein (CRP) < 1 mg/L, albumin 40 g/L) and adequate graft function (serum creatinine 91 umol/L). A recent abdominal CT scan did not reveal any abnormalities compatible with the diagnosis of EPS although a “feces sign”, consisting of intraluminal feculent material in the small bowel, was observed (Figure 1). Additionally, the CT scan showed a small amount of localized ascites in the abdomen but no other diagnostic signs of EPS like bowel tethering, calcifications, or peritoneal thickening. Despite the absence of typical findings, but the severity of the progressive symptoms we considered the possibility of localized EPS, a condition in which the peritoneum shows encapsulating sclerosis predominantly at the level of the terminal ileum [13,14]. Surgical exploration was considered as a final diagnostic procedure. During the operation, a classical picture of EPS was found characterized by a thin cocoon-like sclerotic membrane encasing the small bowel (Figure 2A). A complete resection of the encapsulating sclerotic membrane and total enterolysis were performed, combined with complete removal of the thickened visceral peritoneal membrane (Figure 2B), which lead to restoration of peristalsis durante operationem. Histologic evaluation of the visceral peritoneal membrane was performed, supporting the diagnosis of EPS, and showed dense sclerosis with patchy mononuclear cell infiltration (Figure 3). On the fifth postoperative day, an emergency ileocecal resection with protective loop ileostomy was necessary because of a perforation at the level of the ileocecal junction where a serosal injury had been made during the first operation. The patient recovered soon after surgery, and in an attempt to prevent further recurrences, was prescribed 10 mg twice-daily tamoxifen and 10 mg once daily prednisolone. After five weeks the loop ileostomy was closed and bowel continuity restored, with an uneventful postoperative course. One year after EPS surgery, the patient has gained almost 20 kilograms in body weight and is doing well with a stable graft function and without clinical signs of EPS recurrence.

## Discussion

We report a case of late-onset EPS in a patient presenting 33 months after KTX, with the diagnosis being suspected only on basis of her history of PD and progressive symptoms of intestinal obstruction.

The risk of developing EPS primarily depends on the duration of PD, however, EPS occurs more frequently in patients discontinuing PD treatment [9,15]. In this respect, post-transplantation EPS has gained attention in recent years. The timeline from KTX until diagnosis of EPS varies widely from just a few months to over 2 years. However, most cases of post-transplantation EPS develop within the relatively short time period of one year after transplantation [4,5,9,10].

The current leading theory of EPS pathophysiology assumes that a second hit on an inflamed peritoneal membrane may play an important role in the development of EPS [2]. In line with this theory, cessation of PD treatment causing accumulation of inflammatory and fibrotic mediators [16] may serve as a “second hit” in the development of post-transplantation EPS. In addition, the time from KTX until final EPS diagnosis in our patient was relatively long while patient received tacrolimus monotherapy, and therefore in support of the concept that transplantation per se or exposure to a profibrotic calcineurin inhibitor may have played a role in the development of EPS.

Reaching a definite diagnosis of post-transplantation EPS can be difficult, as is illustrated by our patient, due to aspecific symptoms of bowel obstruction and absence of typical radiological findings. Classically, EPS has an initial inflammatory phase with elevated markers of inflammation, such as CRP [2,11,17]. In this case the patient presented with a non-elevated CRP level. This finding may be explained by the fact that EPS in this patient was detected in its final stage in which the process of inflammation subsided and only a cocoon-like thickened peritoneal membrane remained.

The CT scan aids in the diagnosis of EPS, and according to Tarzi and colleagues may reveal characteristic findings such as peritoneal calcifications, peritoneal thickening, bowel tethering and ascites [12]. In our patient, only a “feces sign [18]” was detected, which may indicate a stenosis in the small bowel, but no classic signs of EPS were found. The thin nature of the sclerotic membrane that covered the bowels apparently prevented detection by CT scan and could only be detected during surgical exploration. The final diagnosis in our patient was made during an exploratory laparotomy, indicating that a low threshold for surgical exploration is needed in case of high clinical suspicion and negative findings on the CT scan. Additionally, surgical exploration can offer a therapeutic benefit as soon as EPS is confirmed, as peritonectomy and enterolysis can be performed during the same operation.

Although steroids and tamoxifen have shown to be of benefit in the treatment of EPS, this may only be the case in the early stage of active disease that is characterized by elevated markers of inflammation [11]. The ultimate treatment of EPS is surgery, involving resection of the sclerotic membrane. Our patient developed a peritonitis on the fifth day after surgery due to a perforation at the ileocecal junction, based on a single serosal injury that was made during the initial operation, and which was sutured straight away. This underscores the hazards of EPS surgery, which is a challenging and time consuming procedure, and preferably should be performed in a specialized center [19]. In experienced hands, elective EPS surgery has a mortality rate <10% [20], however, this may be much higher in patients with an acute indication. Recurrence rates after surgery are high and reported to be 25% in some studies [21]. Our patient received tamoxifen and prednisolone until one year after surgery. Although the benefits of tamoxifen have not been clearly determined, it is believed to have antifibrotic properties [1] and therefore may be useful in preventing recurrences.

## Conclusion

In conclusion, EPS may occur several years after transplantation in the absence of systemic inflammation and typical radiological abnormalities. Obtaining an accurate diagnosis of post-transplantation EPS is challenging, however, a low threshold for surgical exploration in case of high clinical suspicion and without positive findings on the CT scan is mandatory. Surgical treatment for EPS by an experienced surgeon may be indicated to restore bowel movement, improve nutritional status, and alleviate symptoms. Furthermore, surgical exploration in a specialized center should be considered earlier rather than later when EPS is suspected. Finally, this case underscores the important value of considering a possible diagnosis of late-onset EPS even several years after KTX in patients with a long duration of pre-transplant PD.

## Consent

Written informed consent was obtained from the patient for publication of this Case report and any accompanying images. A copy of the written consent is available for review by the Editor of this journal.

## Competing interests

The authors declare that they have no competing interest.

## Authors’ contributions

SMH(1) and MGHB designed and drafted the main manuscript. SMH(1), FJMFD, MRK, SMH, and MGHB have made contributions to the case report by participating in the interpretation of patient data and revision of the final draft. MGHB and FJMFD were closely involved in patient care. All authors have read and approved the final manuscript.

## Pre-publication history

The pre-publication history for this paper can be accessed here:

http://www.biomedcentral.com/1471-2369/14/203/prepub
